# Four-Year Evolution of a Thrombophylaxis Protocol in an Enhanced Recovery After Surgery (ERAS) Program: Recent Results in 485 Patients

**DOI:** 10.1007/s11695-018-3299-4

**Published:** 2018-05-12

**Authors:** Marie-Cécile Blanchet, Vincent Frering, Benoît Gignoux, Yann Matussière, Philippe Oudar, Romain Noël, Alban Mirabaud

**Affiliations:** 1 Clinique de la Sauvegarde, Lyon, France; 2 Espace Médico-Chirurgical, Immeuble “Trait d’Union” - Entrée A29, Av des Sources, 69009 Lyon, France; 3 Department of anesthesiology, Clinique de la Sauvegarde, Lyon, France

**Keywords:** Enhanced recovery after surgery, Bariatric, Omega loop, Sleeve gastrectomy, ERAS, MGB, LSG

## Abstract

“Enhanced recovery after surgery” (ERAS) protocols may reduce morbidity, length of hospital stay (LOS), and costs. During the 4-year evolution of a bariatric ERAS protocol, we found that administration of thrombophylaxis selectively to high-risk morbidly obese patients (assessed postoperatively by Caprini score ≥ 3) undergoing omega loop gastric bypass (“mini” gastric bypass) or sleeve gastrectomy resulted in safe outcomes. Both procedures proved equally effective with this protocol. The vast majority of rapidly mobilized, low-risk patients did not appear to require antithrombotic heparin. Similar to other reported ERAS outcomes, our recent year’s results in 485 patients included a mean LOS of 1.08 ± 0.64 days (range 1–14), with 460 (95.0%) discharged on day 1 and 99.6% by day 2. There were 13 30-day complications (2.7%), two reinterventions (0.4%), and no hemorrhages.

## Introduction

“Enhanced recovery after surgery” (ERAS) programs [1, 2], as opposed to “fast-track” postoperative protocols, are designed to provide a comprehensive pre- *and* postoperative clinical pathway that optimizes anesthesia and analgesia, limits surgical strain, facilitates early mobilization, and augments nutritional effectiveness. Several bariatric ERAS studies, including one randomized trial [3] and three meta-analyses [4–6], have reported patient safety equivalent to standard postoperative protocols while achieving more rapid mobilization, briefer lengths of stay (LOS), decreased costs, and a faster return to normal activities [1, 7–10]. Recent bariatric ERAS and fast-track studies report that anticoagulants are often administered prophylactically to all bariatric patients, and in higher doses to those with a body mass index (BMI) ≥ 40 kg/m^2^ [11–14]. The all-inclusive approach was confirmed as the norm in a 2017 systematic review and guideline published by the European Venous Thromboembolism Prophylaxis Task Force, in which a paucity of high-quality randomized controlled trials precluded their providing strong, specific thrombophylaxis recommendations [15].

During the 4-year development of our center’s bariatric ERAS protocol, we found that the use of thrombophylaxis selectively, only in morbidly obese postoperatively high-risk patients, was effective in ensuring safe outcomes while achieving more rapid mobilization [16]. The program’s evolution began in 2012 with a feasibility study of same-day discharge for patients undergoing laparoscopic adjustable gastric banding (LAGB). In this study, 6.5% of patients were successfully ambulatory with a low readmission rate (3/86, 3.5%). In 2013, we offered outpatient colectomy, publishing the results of this procedure in 2015 (LOS < 12 h, *n* = 20) [17]. In early 2017, we reported outcomes of a prospective patient series begun in 2012 (*n* = 374) that incorporated a bariatrics-specific ERAS protocol, the first study of ERAS in morbidly obese patients undergoing omega loop gastric bypass (“mini” gastric bypass, MGB) vs laparoscopic sleeve gastrectomy (LSG) [16]. Time in the operating room was < 45 min, and mean LOS was 1.2 days, a briefer LOS than that reported in a meta-analysis of ERAS with bariatric surgery [6]. In 86.0% of patients, patients were discharged on day 1, with 96.9% discharged by day 2. Postoperative complications were low, 2.9%, with readmission in 2.1%, and reintervention in 1.3%. The ERAS program was equally successful in MGB and LSG. However, near the beginning of the series, several postoperative cases of hemorrhage were noted. We elected to stop administering postoperative low molecular-weight heparin (LMWH) routinely to all patients, and use it instead only in the subsequent patients with a Caprini Deep Vein Thrombosis (DVT) score of ≥ 3 [18].

The success of the adjusted protocol in the remainder of our original bariatric series appeared to confirm that postoperative LMWH was not necessary in patients at low risk of DVT (Caprini score < 3) [16]. Based on these findings, the current follow-on study was designed to assess outcomes in a prospective MGB and LSG cohort systematically treated using our bariatric ERAS protocol with the revised LMWH decision tree.

## Methods

### Patient Inclusion

Patients ≥ 18 years old who met the accepted international criteria for bariatric surgery (i.e., US National Institutes of Health 1991 Guidelines, International Federation for the Surgery of Obesity (IFSO) standards) were included in this prospective cohort study. The ERAS clinical protocol was explained to patients by the surgeon in detail. Approval for inclusion in the study and written formal consent was obtained from each patient.

### Bariatric ERAS Program

During the bariatric ERAS study, a nurse followed each patient personally. Patients were encouraged to be active agents of their own well-being instead of passive recipients of a surgical process. The detailed process of patients moving through the preoperative, operative, and postoperative ERAS protocol and follow-up, including the MGB and LSG procedures performed, and anesthesia and analgesia employed, has been described in detail in our original bariatric ERAS publication [16].

### Postoperative Thrombophylaxis

At the outset of our prior, original bariatric ERAS study, prophylactic LMWH was administered systematically to all patients. Due to the unsatisfactory development of several cases of postoperative hemorrhage, we altered the anticoagulation protocol in subsequent patients, with positive results: Incidence of hemorrhage was not increased in either MGB or LSG patients with a Caprini DVT score < 3 who, thus, did not receive LMWH [16]. Only those with a Caprini score ≥ 3 received LMWH thrombophylaxis from postoperative day 1 through day 10. The use of the revised ERAS thrombophylaxis decision tree, based on a Caprini score as determined post procedure in the operating room by the surgeon and anesthesiologist, is the basis for the current, prospective study of MGB and LSG.

### Statistical Analysis

Analyses were performed using the SPSS statistical package (version 20; IBM, Chicago, IL). The independent samples *t* test was employed to analyze between-group differences in continuous variables. Categorical data differences were analyzed with Fisher’s exact test. The statistical tests used were two tailed, and statistical significance was set at *p* < 0.05.

## Results

Between January 1, 2016, and December 31, 2016, 485 patients (84.7% female, mean age 40.0 ± 11.2 years [range18–67], mean BMI 40.5 ± 4.5 [27–55]) underwent bariatric surgery in association with our revised ERAS protocol. Nearly half (42.5%) reported having had a previous LAGB. More than 70.0% presented with at least one comorbidity, including 59.4% with articular disease, 19.2% hypertension, 11.1% hyperlipidemia, 11.3% obstructive sleep apnea, and 8.3% type 2 diabetes mellitus. Five percent of patients reported using tobacco products regularly.

Of the 485 patients, 244 (50.3%) underwent MGB and 241 (49.7%) underwent LSG. A significantly greater percentage of females comprised the MGB group (89.8 vs 79.7%, *p* < 0.05) (Table 1). MGB was the operation of choice by a narrow margin (53.3%) in females, whereas LSG was the operation of choice for most males (66.2%). LSG patients were significantly younger, heavier, and more likely to use tobacco. Patients undergoing MGB were significantly more likely to have undergone a previous LAGB.

**Table 1:** Preoperative patient characteristics and postoperative outcomes for MGB and LSG patients in an “enhanced recovery after surgery” (ERAS) program

Variable	MGB (*n* = 244)	LSG (*n* = 241)	*p* value
Preoperative			
Female, *n* (%)	219 (89.8)	192 (79.7)	< 0.05*
Age (years)	43.1 *±* 10.7 (19–67)	36.8 *±* 10.8 (18–63)	< 0.05^†^
Height (cm)	163.6 *±* 7.4 (147–183)	165.5 *±* 8.4 (148–185)	< 0.05^†^
Absolute weight (kg)	107.1 *±* 18.5 (75–164)	113.0 *±* 15.9 (80–172)	< 0.05^†^
Body mass index (kg/m^2^)	39.9 *±* 4.8 (28–52)	41.1 *±* 4.3 (27–55)	< 0.05^†^
Previous LAGB, *n* (%)	161 (66.0)	45 (18.7)	< 0.05*
Articular disease, *n* (%)	136 (55.7)	152 (63.1)	0.12*
Hypertension, *n* (%)	49 (20.1)	44 (18.3)	0.66*
Type 2 diabetes, *n* (%)	26 (10.7)	14 (5.8)	< 0.07*
Hyperlipidemia, *n* (%)	21 (8.6)	33 (13.7)	0.08*
Sleep apnea, *n* (%)	22 (9.0)	33 (13.7)	0.12*
Steatosis, *n* (%)	40 (16.4)	34 (14.1)	0.53*
Tobacco use, *n* (%)	7 (2.9)	17 (7.1)	< 0.05*
Caprini ≥ 3, *n* (%)	10 (4.1)	9 (3.7)	1.00*
Operative			
Mean operative time (min)	43.3 *±* 11.7 (32.0–57.0)	33.1 *±* 9.8 (25.0–55.0)	< 0.05^†^
Length of hospital stay (days)	1.10 *±* 0.9 (1–14)	1.05. *±* 0.3 (1–4)	0.41^†^
Postoperative			
Complications	5 (2.1)	8 (3.3)	0.41*
Hemorrhage	0 (0.0)	0 (0.0)	1.00*
Portal vein thrombosis	0 (0.0)	3 (1.2)	0.12*
Occlusion/trocar	1 (0.4)	1 (0.4)	1.00*
Colitis	2 (0.8)	2 (0.8)	1.00*
Fistula	1 (0.4)	0 (0.0)	1.00*
Parietal hematoma	1 (0.4)	1 (0.4)	1.00*
Vomiting	0 (0.0)	1 (0.4)	1.00*
Rehospitalization	4 (1.6)	7 (2.9)	0.38*
Reinterventions	1 (0.4)	1 (0.4)	1.00*

*Fisher’s exact test

^†^Independent samples *t* test

**Fig. 1:**
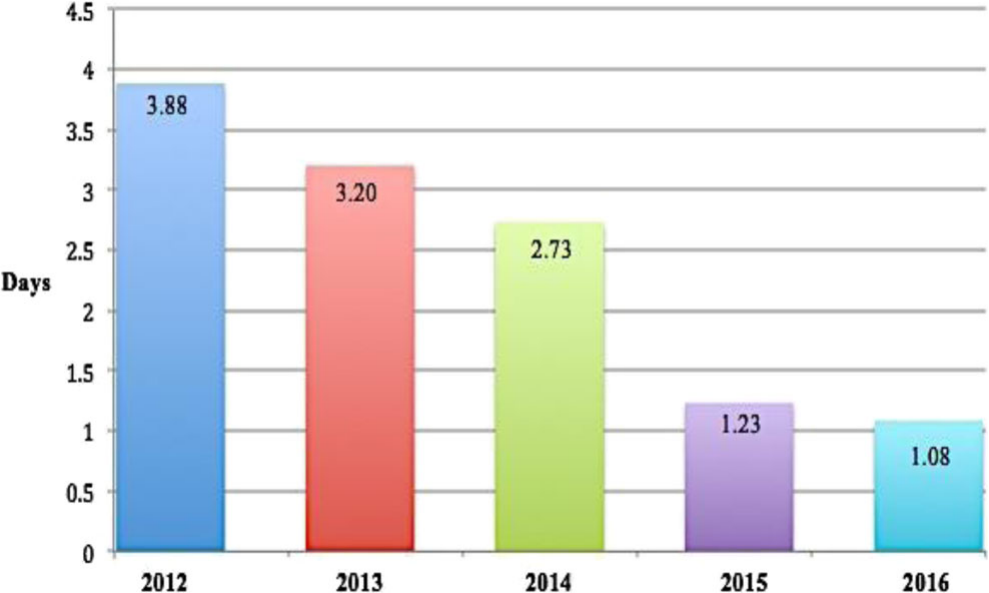
Evolution of trends in mean length of stay (LOS) of our center’s bariatric ERAS series from 2012 to 2016

Overall, mean operative time was 38.0 ± 11.1 min, and mean LOS for all operative procedures was 1.08 ± 0.64 days (1–14). Specifically, 460 patients (95.0%) were discharged on day 1, and by day 2, 23 additional patients were discharged (total 483, 99.6%). Only 2 patients (0.4%) had primary hospital stays that exceeded 2 days. Figure 1 shows the evolution of trends in the mean LOS of our center’s complete bariatric ERAS series, from 2012 to 2016.

Generally, those hospitalized for 2 or more days were treated for minor complications. There was no mortality, pulmonary embolism, or hemorrhage. Overall, only 19 patients (3.9%) presented with Caprini scores ≥ 3 and were thus administered LMWH beginning on postoperative day 1 through day 10. The Caprini score proved to be a successful indicator of thromboembolic risk. Also, interestingly, LMWH patients were equally distributed between the two surgical groups. Although a systematic review by Becattinni et al. (2011) reported an LMWH regimen with a mean of 15 days, we found 10 days of thrombophylaxis provided safety and effectiveness, perhaps since we encourage our patients to be quite active in accord with the ERAS protocol [19].

With the exception of a difference of 10.2 min in mean operative time, there were no statistically significant differences between the outcomes of the two bariatric procedures performed with the ERAS protocol (Table 1**)**. Mean operating time for MGB vs LSG was 43.3 *±* 11.7 vs 33.1 *±* 9.8 min (*p* < 0.05). Mean LOS for MGB vs LSG patients was 1.10 *±* 0.9 vs 1.05 *±* 0.3 days (*p* = 0.41). Overall, 13 patients (2.7%) experienced 30-day complications (MGB 5; LSG 8; 2.1 vs 3.3%, *p* = 0.41). Eleven patients (2.3%) required rehospitalization (MGB 4; LSG 7; 1.6 vs 2.9%, *p* = 0.38). Two patients (0.41%) required reintervention (MGB 1; LSG 1; 0.4 vs 0.4%, *p* = 1.00).

Three patients (0.6%) experienced portal vein thrombosis (MGB 0; LSG 3; 0.0 vs 1.2%, *p* = 0.12). This was the only measured outcome (likely due to anatomical reasons) where differences in complication rates between procedures slightly approached significance. Two patients (0.4%) suffered orifice occlusion at the trocar site, 1 with MGB, 1 with LSG (0.4 vs 0.4%; *p* = 1.00). Also, 4 patients (0.8%) experienced colitis, 2 with MGB, 2 with LSG (0.8 vs 0.8%; *p* = 1.00). One patient (0.2%) developed a fistula (MGB 1; LSG 0; 0.4 vs 0.0%; *p* = 1.00) prior to discharge. The post-MGB fistula was treated conservatively with an endoscopic pigtail drain; this patient experienced the longest LOS in the series (14 days). Two patients experienced a parietal hematoma, 1 with MGB, 1 with LSG (0.4 vs 0.4%; *p* = 1.00).

At 1 month, there was 95.0% follow-up; 24 patients (5.0%) failed to attend the first postoperative consultation. Overall patient satisfaction was high: 98.6% reported that they “were satisfied with this clinical pathway,” and “they would recommend it to others.”

## Conclusion

In the current 1-year study of 485 morbidly obese patients undergoing bariatric surgery, the ERAS program employed was safe and effective with MGB and LSG. Mean LOS was less than the reported meta-analytic mean LOS for bariatric ERAS programs. The great majority of patients were discharged on day 1, and the remainder by day 2. Postoperative complications, readmissions, and reinterventions were low. The bariatric ERAS program with revised thrombophylaxis protocol stratifying administration of postoperative thrombophylaxis based on Caprini DVT risk level appeared to be safe and effective.

Obesity itself is a well-defined demographic risk factor for DVT; research suggests no increased risk of major hemorrhage with the use of prophylactic agents in bariatric surgery patients. Therefore, painstaking judgment must be used when opting not to use chemical prophylaxis against the potentially fatal complication of bleeding, particularly when the side effects of thrombophylaxis are minimal. Yet, minimizing complications due to bleeding from chemical thrombophylaxis is also a desired outcome. Thus, we recommend reduction or elimination of postoperative bariatric thrombophylaxis only under conditions identical to those employed in our tested and evolved ERAS protocol that features consultation in the operating room between both anesthesiologist and surgeon according to the Caprini score decision tree, and close daily patient follow-up for 10 days by a protocol-trained nurse. Randomized controlled studies of ERAS in bariatric surgery vs standard care are needed.
